# Effects of a metabotropic glutamate receptor subtype 7 negative allosteric modulator in the periaqueductal grey on pain responses and rostral ventromedial medulla cell activity in rat

**DOI:** 10.1186/1744-8069-9-44

**Published:** 2013-09-03

**Authors:** Enza Palazzo, Ida Marabese, Livio Luongo, Serena Boccella, Giulia Bellini, Maria Elvira Giordano, Francesca Rossi, Mariantonietta Scafuro, Vito de Novellis, Sabatino Maione

**Affiliations:** 1 Department of Anaesthesiology, Surgery and Emergency, The Second University of Naples, Piazza Luigi Miraglia 2, Naples 80178, Italy; 2 Department Experimental Medicine, The Second University of Naples, via Costantinopoli 16, Naples 80138, Italy; 3 Department of Woman, Child and General and Specialistic Surgery, The Second University of Naples, Naples 80138, Italy

**Keywords:** Metabotropic glutamate receptor subtype 7, Spare nerve injury, Ventrolateral periaqueductal grey, Rostral ventromedial medulla, ON and OFF cells, Formalin test

## Abstract

The metabotropic glutamate receptor 7 (mGluR7) negative allosteric modulator, 6-(4-methoxyphenyl)-5-methyl-3-pyridin-4-ylisoxazolo[4,5-c]pyridin-4(5H)-one (MMPIP), was locally microinjected into the ventrolateral periaqueductal gray (VL PAG) and the effect on pain responses in formalin and spare nerve injury (SNI) -induced neuropathic pain models was monitored in the rat. The activity of rostral ventromedial medulla (RVM) “pronociceptive” ON and “antinociceptive” OFF cells was also evaluated. Intra–VL PAG MMPIP blocked the first and second phase of nocifensive behaviour in the formalin pain model. MMPIP increased the tail flick latency and simultaneously increased the activity of the OFF cells while inhibiting that of ON cells in rats with SNI of the sciatic nerve. MMPIP failed to modify nociceptive responses and associated RVM ON and OFF cell activity in sham rats. An increase in mGluR7 gene, protein and staining, the latter being associated with vesicular glutamate transporter-positive profiles, has been found in the VL PAG in SNI rats. Blockade of mGluR7 within the VL PAG has an antinociceptive effect in formalin and neuropathic pain models. VL PAG mGluR7 blockade offers a target for dis-inhibiting the VL PAG-RVM pathway and silencing pain in inflammatory and neuropathic pain models.

## Background

Metabotropic glutamate subtype receptor 7 (mGluR7) is the most highly conserved [1] and widely distributed among mGluRs, suggesting a critical role in regulating excitatory synaptic transmission in the central nervous system (CNS) [2-5]. It is mainly located in the active presynaptic cleft of the glutamatergic synapse where it acts as autoreceptor [6-10] or as hetereoreceptor controlling the release of neurotransmitters other than glutamate [7,11]. The characterization of the functional role of mGluR7 in the CNS has been hampered by the lack of selective agents and limited to mGluR7 knockout mouse studies until valuable pharmacological tools for studying its function were developed: the N,N’-dibenzyhydrylethane-1,2-diamine (AMN082), a highly selective positive allosteric modulator (PAM) [12] and the 6-(4-methoxyphenyl)-5-methyl-3-(4-pyridin)-4ylisoxazolo[4,5-c]pyridin-4(5H)-one (MMPIP) a negative allosteric modulator (NAM) which exhibits intrinsic inverse agonist activity [13,14]. AMN082 and MMPIP have been shown to penetrate the blood–brain barrier in vivo [12,15].

Periaqueductal grey (PAG) is a key supraspinal site of the antinociceptive descending pathway which includes the rostral ventromedial medulla (RVM) and the dorsal horn of the spinal cord. PAG control of pain is produced concomitantly with the modulation of pain-responding neurons of the RVM: the ON cells which are activated and OFF cells which are inhibited by nociceptive stimuli [16,17]. These cells also respond differently to centrally acting analgesics: μ-opioid or CB1 receptor agonists depress ON cell activity while they increase that of OFF cells [16,18]. Neutral cells, another class of neurons found in the RVM, are instead unaffected by noxious stimuli and analgesic agents. mGluR7 stimulation by AMN082 has been shown to facilitate pain behavior when microinjected into the ventrolateral periaqueductal grey (VL PAG) [19] and in the central nucleus of the amygdala (CEA) [20]. The pain facilitatory effect due to mGluR7 stimulation in the VL PAG was associated with consistent changes in the RVM cell activity [19]. AMN082 also slightly reduced cold and mechanical allodynia in neuropathic mice [21] when systemically administered and inhibited cardiac nociception when administered in the nucleus tractus solitarius (NTS) [22]. Blockade of mGluR7 by systemic MMPIP has proven not to change nociceptive thresholds in the tail immersion test or in the first and second phase of the formalin test [15]. Moreover, mGluR7 expression has been proven to decrease in the lumbar dorsal horn of mice with a neuropathic pain condition [21], but has never been evaluated in the PAG. Thus the role of mGluR7 in pain perception is still far being established and in particular the effect of the blockade of mGluR7 receptor at PAG level in healthy and chronic pain conditions has never been investigated. In this study we therefore microinjected MMPIP into the VL PAG and evaluated the effect on: i) nocifensive responses induced by a peripheral injection of formalin; ii) electrophysiological changes in the RVM ON and OFF cell activity and associated tail flick responses in a model of neuropathic pain induced by the spared nerve injury (SNI) of the sciatic nerve; iii) changes of mGluR7 expression in the VL PAG of SNI rats.

## Results

### Effects of intra- VL PAG MMPIP on formalin-induced nocifensive behavior

**Figure 1:**
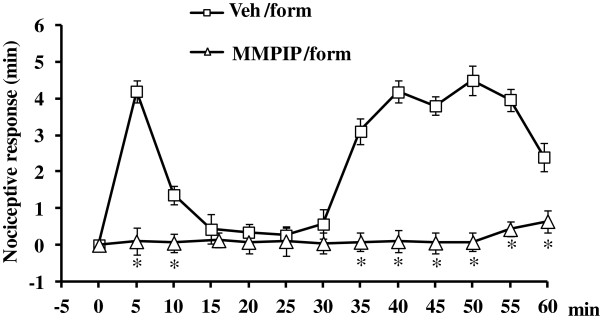
**Time course of the nociceptive behaviour induced by subcutaneous formalin (5% formalin, 50 μl, form) injected 10 min after the intra-VL PAG microinjection of vehicle (0.05% DMSO in ACSF, 50 μl, Veh) or MMPIP (5 μg/0.2 μl).** Recording of nociceptive behaviour began immediately after the injection of formalin (time 0) and was continued for 60 min. Each point represents the total time spent lifting or licking the injected paw (mean ± SEM). Time measurements were taken every 5 min. Each group comprised 6–8 rats. * indicate significant differences vs rats receiving intra-VL PAG ACSF and formalin into the dorsal surface of the hind paw. P < 0.05 was considered statistically significant.

Formalin-induced nociceptive behavior was quantified by calculating the amount of time that the rats spent lifting and/or licking the formalin-injected hind paw. Control rats receiving the subcutaneous injection of saline (0.9% NaCl) into the hind paw did not display any nociceptive behavior (n = 6, not shown). Subcutaneous injection of formalin in rats receiving intra-VL PAG vehicle resulted in a typical biphasic nociceptive response. The first phase was characterized by a first robust nociceptive response followed by a transient decline thereafter. The second phase started 30 min after formalin reaching a peak at 50 min (Figure 1). This nocifensive behaviour did not differ from that induced by the peripheral injection of formalin alone. The intra-VL PAG microinjections of MMPIP (5ug/0.2ul) significantly reduced nocifensive responses in the first [F(2,14) = 179.66, P < 0.01, mixed design two-way ANOVA versus rats receiving the intra-VL PAG microinjection of vehicle and the subcutaneous injection of formalin into the hind paw] and second phase [F(2,14) = 120.77, P < 0.01), mixed design two-way ANOVA versus rats receiving the intra-VL PAG microinjection of vehicle and the subcutaneous injection of formalin into the hind paw] of the formalin test, as recorded 5 and 50 min after formalin peripheral injection (Figure 1).

### Effect of intra-VL PAG microinjection of MMPIP on tail flick latencies in sham and SNI rats

**Figure 2:**
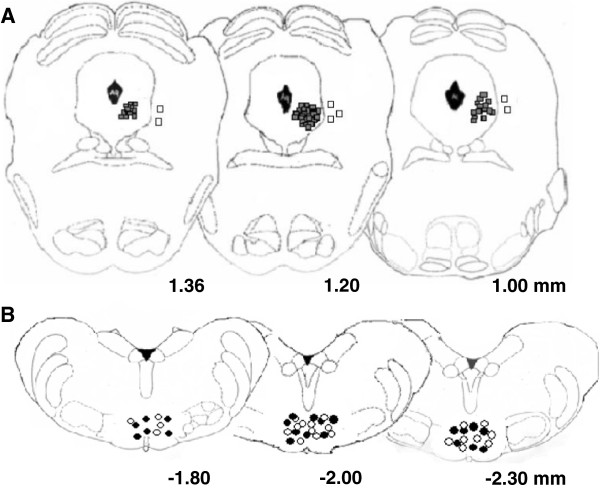
**Representative schematic illustration of the microinjection sites for drug administration into the VL PAG (A) and electrode positions within RVM (B).** Coronal brain slices containing the VL PAG and recording sites into the RVM were processed after the experiment for histological analysis. The numbers above the illustration show the distance from the bregma (Paxinos and Watson, 1998). Black squares indicate microinjection site tips into the VL PAG and white squares those intentionally performed outside the PAG for placement controls **(A)**. Circles indicate electrode tips in the RVM, in particular white circles represent ON cells and black circles the OFF cells with overlapping tip positions indicated with a single symbol **(B)**. RMn, RGcn and RGcnα refer to nucleus raphe magnus, nucleus reticularis paragigantocellularis, nucleus reticularis gigantocellularis pars α, respectively.

**Figure 3:**
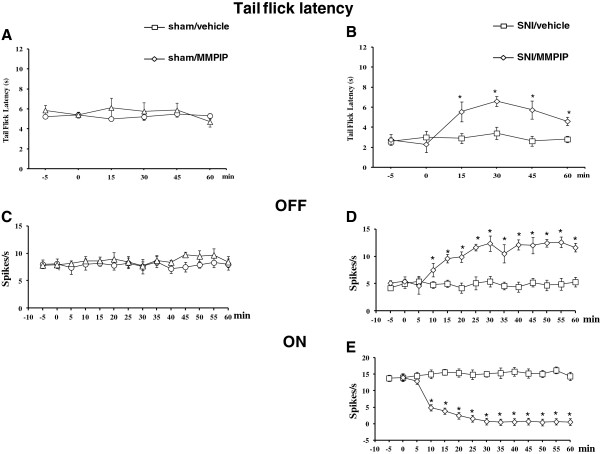
**Effects of intra-VL PAG microinjections of vehicle (0.05% DMSO in ACSF) or MMPIP (5 μg/0.2 μl) on tail flick latencies (A and B) and the spontaneous firing of RVM OFF (C and D) and ON cells (E) in sham (A and C) and SNI (B, D and E) rats 14 days after surgery.** Each point represents the mean ± S.E.M of 12–14 **(A** and **B)** or 6–7 **(C**, **D** and **E)** rats per group. * indicates statistically significant difference vs pretreatment values. P values < 0.05 were considered statistically significant.

Tail flicks were elicited every 5 min for 15 min prior to microinjecting MMPIP (5 μg/0.2 μl) or respective vehicle into the VL PAG. Only rats whose microinjection site was located within the VL PAG (black squares) were used for data computation. Cannulae were also intentionally implanted 1 mm outside from VL PAG for control experiments verifying the specificity of the area-induced effects (white squares, n = 7) (Figure 2A). In SNI rats, latency to the tail flick was significantly lower compared to that of the shams [F(2,24) = 6.62, P < 0.05, mixed design two-way ANOVA versus sham rats]. Intra-VL PAG microinjection of vehicle did not change tail flick latency in sham and SNI rats compared to pre-treatment values. Intra-VL PAG microinjection of MMPIP (5 μg/0.2 μl) significantly increased the tail flick latency compared to pretreatment values in SNI rats (P < 0.05, n = 12, paired t test) (Figure 3B) but was devoid of any activity in sham rats (Figure 3A). MMPIP (5 μg/0.2 μl) was also intentionally microinjected 1 mm outside the VL PAG (n = 7), where it failed to change tail flick latency (not shown).

### Effect of intra-VL PAG microinjection of MMPIP on the ongoing activity of RVM ON and OFF in sham and SNI rats

In SNI rats, the population of OFF cells had a lower frequency of spontaneous activity compared to that of the shams **[**F(2,12) = 10.56, P < 0.001, mixed design two-way ANOVA versus sham rats]. Microinjection of vehicle (0.05% DMSO in ACSF) did not change the spontaneous activity of the OFF cells compared to pretreatment values in sham and SNI rats (Figure 3C and D). In sham rats, the intra-VL PAG microinjection of MMPIP (5 μg/0.2 μl) did not change the ongoing OFF cell activity (Figure 3C). In SNI rats, intra VL-PAG microinjections of MMPIP (5 μg/0.2 μl) caused a significant increase in the spontaneous firing activity of the OFF cells compared to pretreatment values (P < 0.05, n = 6, paired t test) (Figure 3D). In sham rats, the neurons identified as ON cells by a burst of activity just before tail flick responses were devoid of spontaneous activity in the majority of cases. In SNI rats, all the ON cells encountered displayed spontaneous activity and the intra-VL PAG microinjections of MMPIP (5 μg/0.2 μl) caused a significant decrease in the spontaneous firing compared to pretreatment values (P < 0.05, n = 6, paired t test) (Figure 3E).

### Effect of intra-VL PAG microinjection of MMPIP on tail flick-related ON and OFF cell activity in sham and SNI rats

**Figure 4:**
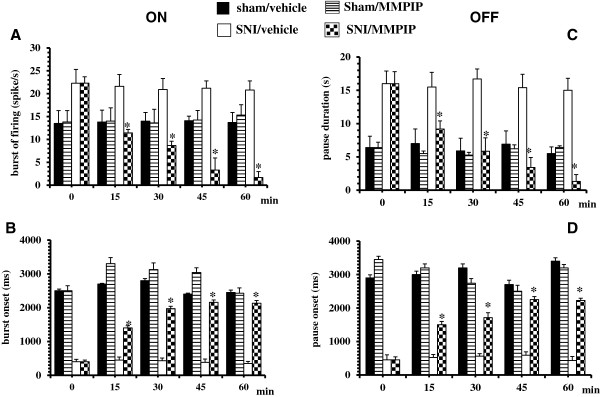
**Effects of intra-VL PAG microinjections of vehicle (0.05% DMSO in ACSF) or MMPIP (5 μg/0.2 μl) on tail flick-evoked burst of firing (A) and onset of the burst (B) of the ON cells or tail flick-evoked pause (C) and onset of the pause (D) of the OFF cells in sham and SNI rats 14 days after surgery.** Each histogram represents the mean ± S.E.M of 6–7 neurons of different treated groups of rats. * indicates significant differences vs pretreatment values. P values < 0.05 were considered statistically significant.

**Figure 5:**
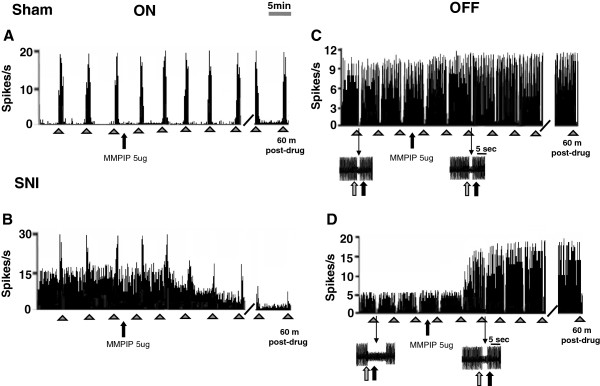
**Examples of ratemater records which illustrate the effect of intra-VL PAG microinjection of MMPIP (5 μg/ 0.2μl) on either the ongoing or tail flick-related burst of activity of identified RVM ON cells (A and B) and ongoing or tail flick-related pause of identified RVM OFF cells (C and D) in sham and SNI rats.** Intra-VL PAG microinjection of MMPIP (5 μg/0.2μl) did not change the ongoing activity and the tail flick-related burst of the ON cell in sham rats **(A)** but reduced them in SNI rats **(B)**. The same treatment did not modified the OFF cell activity in the sham rats **(C)** but increased the ongoing activity and reduced the tail flick-related pause of the OFF cell in SNI rats **(D)**. Scales bars indicate 5 min and small full arrows indicate the noxious stimulation application.

In SNI rats, the population of ON cells had a tail flick-induced burst of firing frequency that was significantly higher compared to the sham rats [F(2,12) = 8.80, P < 0.001, mixed design two-way ANOVA versus sham rats]. The onset of the burst was significantly lower in SNI rats compared to the shams [F(2,12) = 644.1, P < 0.001, mixed design two-way ANOVA versus sham rats]. The mean of the frequency and the onset of the ON cell burst in the sham rats did not differ from healthy rats. The population of the OFF cells in SNI rats had a significantly longer pause [F(2,12) = 19.9, P < 0.001, mixed design two-way ANOVA versus sham rats] and a significantly shorter onset of pause [F(2,12) = 348.04, P < 0.001, mixed design two-way ANOVA versus sham rats]compared to the shams. The duration and onset of the OFF cell pause in the sham rats did not differ from healthy rats. Microinjections of vehicle in sham and SNI rats did not change the tail flick-induced ON cell burst nor the onset of burst or the OFF cell pause or the onset of the pause compared to pretreatment values. Intra-VL PAG microinjection of MMPIP (5 μg/0.2 μl) caused a decrease in both ON cell burst (P < 0.05, n = 6, paired t test) and OFF cell pause (P < 0.01, n = 5, paired t test) (Figure 4A and C) compared to pre-treatment values in SNI rats. Intra VL-PAG microinjection of MMPIP (5 μg/0.2 μl) also caused an increase in both the onset of ON cell burst (P < 0.01, n = 5, paired t test) and the onset of OFF cell pause (P < 0.05; n = 5, paired t test) (Figure 4B and D) compared to pre-treatment values in SNI rats. The same treatment did not change the tail flick-related activity of the ON or OFF cell in sham rats. Representative ratemater records showing tail flick-related activity of ON and OFF cells in sham and SNI rats before and after MMPIP (5 μg/0.2 μl) are shown in Figure 5.

### Gene expression of mGluR7

**Figure 6:**
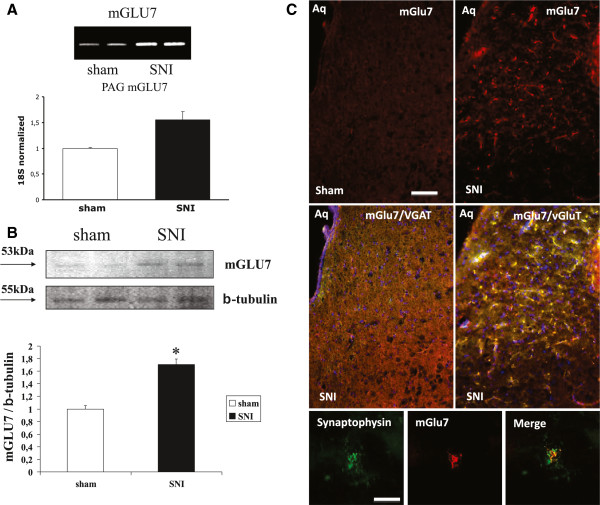
**Expression of mGluR7 mRNA in the VL PAG: RT-PCR analysis starting from 100ng mRNA shows an increase for mGluR7 gene expression in SNI rats compared to sham rats.** Quantification of the expression levels is reported in the graphic that shows RT-PCR data value for mGluR7 relative to the housekeeping 18S gene and normalized with respect to the mean of the shams **(A)**. Western blot analysis for mGluR7 protein in PAG lysate from SNI and sham rats normalized with respect to β-tubulin and sham mean **(B)**. Expression of mGluR7 in the PAG of SNI and sham rats. Double staining shows the high expression of mGluR7 on vGluT positive terminals (right) and low expression on VGAT positive terminals (left). Wave length is 568 nm for red staining and 488 for green staining (not shown) the merge was performed of 488 and 568 channels (yellow). Lowest panel indicate the double staining mGluR7 (red) synaptophysin (green) and relative merge **(C)**. Purple spots indicate a DAPI and red background/fluorescence spot overlapping. Scale bar = 100 μm. Data are represented as a mean ± SEM. p < 0.05 was considered statistically significant.

The semi-quantitative analysis of mRNA levels within the VL PAG measured by RT-PCR amplification showed a significant increase in the mGluR7 gene in SNI rats (Figure 6A)**.**

### Western blotting

Western blot analysis showed a significant increase in mGluR7 protein levels in the VL PAG in rats with SNI (two-tailed unpaired Student’s t-tests, P < 0.01; t = −9.116, n = 10) (Figure 6B).

### Immunohistochemistry

Immunohistochemical analysis revealed a massive increase of staining for mGluR7 in the VL PAG in rats with SNI compared to sham operated rats, confirming the western blotting measurement (Figure 6C). Moreover we found that mGluR7 was mainly expressed by vGluT stained terminals, whereas vGAT positive terminals were not counterstained by mGluR7, assuming the autoreceptor nature of the mGluR7. The predominant presynaptic nature of mGluR7 has been corroborated by the double labelling with synaptophysin, a presynaptic terminal marker.

## Discussion

This study is the first to investigate the role of mGluR7 blockade in the VL PAG on formalin-induced pain behaviour, on tail flick latency and the activity of the RVM ON and OFF cells in control and neuropathic pain conditions in the rat. A subtype-selective negative allosteric modulator for mGluR7 (MMPIP) [13,14] was microinjected into the VL PAG of rats receiving the injection of formalin into the dorsal surface of the hind paw or rats which had undergone the SNI of the sciatic nerve or sham surgery. The key findings are as follows: intra-VL PAG MMPIP inhibited the early and late phases of formalin-induced nocifensive behavior; increased tail flick latency in SNI rats and modified the ongoing and the tail flick evoked activity of the ON and OFF cell of the RVM accordingly. MMPIP was instead ineffective in sham animals.

Our previous studies have shown that mGluR7 stimulation plays a facilitatory role on pain. mGluR7 stimulation by AMN082, a selective mGluR7 agonist [12], locally microinjected into the VL PAG and CeA, reduced the nociceptive threshold [19,20,23]. AMN082 also decreased extracellular glutamate release within the VL PAG [19]. Glutamate into the PAG relieves pain [24-26] thus due to the autoreceptor role of mGluR7 [6,27-30], AMN082 may lead to a decrease in glutamate level and subsequent pain facilitation. We assume that the mGluR7 blockade by MMPIP within the PAG would block this effect (the decrease in glutamate tone) thus dis-inhibiting the antinociceptive descending pathway with consequent inhibition of formalin-induced nocifensive behaviour. A recent study has however shown that MMPIP had no analgesic effect in either the tail immersion test or formalin test in mice when systemically administered [15].

MMPIP has been shown to penetrate into the brain after systemic administration [15] thus the effect of systemically administered MMPIP involves brain, spinal and peripheral mGluR7. The overall effect of systemic mGluR7 blockade may result from a complex balance between pain facilitatory or inhibitory actions that are dependent on mGluR7 localization on presynaptic elements of glutamate or non –glutamate containing neurons [10,19,31,32]. The combination of different/opposite effects could mask the effect of MMPIP on nociceptive responses, determining the lack of effect. A lack of effect of systemically administered mGluR7 agonist, AMN082, has also been shown by Stachowicz et al. [33] in the hot plate test whilst the intrathecal AMN082 administration has been shown to inhibit both the early and late phase of formalin-induced pain behaviour [34].

Another concern of the following study is that the blockade of mGluR7 by MMPIP within the VL PAG has reduced the nocifensive behavior in both the first and second phases of the formalin test. PAG stimulation has been shown to inhibit the early and late phases of formalin-induced nocifensive behavior [35]. By reducing the inhibition of glutamate release through mGluR7 stimulation, intra-VL PAG MMPIP would act as a PAG “indirect stimulator” leading to pain inhibition [36-39]. Moreover, the second phase of formalin test is the result of central neuron changes produced by the neural activity generated during the early phase [40-44], therefore the blockade of the early phase prevents the development of the late phase.

The PAG modulatory effect on pain involves RVM which lies downstream of the PAG and upstream of the dorsal horn spinal cord. Within the RVM, ON cells, which show a burst immediately prior to a nociceptive reflex [14,45,46], have a facilitatory effect, whereas OFF cells, which show a pause prior to a nociceptive reflex, have an inhibitory effect on nociception [16,18,47]. The effect of intra-VL PAG microinjections of MMPIP on the ON and OFF cell activity of RVM has been investigated in sham and SNI rats.

SNI induced marked pronociceptive changes in the ongoing and tail flick-related activity of RVM ON and OFF cells. The spontaneous discharge of the ON cells was increased while that of the OFF cells decreased in the SNI-operated rats. The ON cell burst and the OFF cell pause were increased while the onset of the ON cell burst and of the OFF cell pause proved to be decreased. ON cell hyperactivity and OFF cell hypoactivity following neuropathic pain in the pain descending system contributing to chronic pain symptoms have been reported [48-53]. Intra-VL PAG MMPIP reverted SNI-induced changes in both spontaneous and tail flick-related activities of ON and OFF cells. In particular, it decreased the burst frequency and increased the onset of the burst of "pronociceptive" ON cells. Furthermore, MMPIP increased the spontaneous activity of "antinociceptive" OFF cells, reduced the pause duration and increased the onset of the OFF cell pause in SNI rats.

These effects were consistent with a behavioural anti-allodynic effect monitored simultaneously through tail flick in the SNI rats. An anti-allodynic rather then an antinociceptive effect was suggested also by the fact that intra-VL PAGMMPIP did not show any effect in the shams. mGluR7 stimulation in the VL PAG instead induced opposite effects such as the facilitation of the ON cell activity and the inhibition of OFF cell activity within the RVM [19], consistently with behavioural pain facilitation [54].

mGluR7 is the most widely distributed presynaptic mGluR subtype in the central and peripheral nervous system [31,55]. Its xpression of mGluR7 proved to be significantly down-regulated in dorsal root ganglia after sciatic nerve axotomy [56] and in the spinal dorsal horn after spinal nerve ligation [57]. Changes in expression of mGluR7 in supraspinal areas such as the PAG in chronic pain conditions are yet to be investigated. Upregulation in the expression of mGluR7 gene and protein has been observed in the VL PAG 14 days after neuropathic pain induction. Therefore changes in expression of mGluR7 seem to be site specific and may justify the effect of MMPIP in SNI rats and the lack of any effect in the shams. The immunohistochemistry showed an increased expression of mGluR7 being co-expressed with vGluT1 positive profiles. These increased mGluR7 positive profiles on vGluT1 counterstaining after SNI, indicated that mGluR7 is over-expressed in neuropathic pain conditions on glutamatergic neurons within the VL PAG, confirming that MMPIP would act by dis-inhibiting the VL PAG antinociceptive control.

## Conclusion

The blockade of mGluR7 by microinjection of MMPIP into the VL PAG inhibits nocifensive responses to formalin and reduces thermonociception and ON cell activity, while enhancing OFF cell activity in SNI rats. Intra-VL PAG MMPIP did not affect thermal threshold or RVM ON and OFF cell activity in the shams. Furthermore, mGluR7 proved to be over-expressed on glutamatergic terminals 14 days after SNI. It would therefore appear that intra VL PAG blockade of mGluR7 offers a suitable strategy for activating the antinociceptive pathway and inhibiting pain responses in inflammatory and neuropathic pain conditions.

## Methods

### Animals

Male Sprague–Dawley rats (Harlan, Italy) weighing 250–280 g were housed three per cage under controlled illumination (12 h light/12 h dark cycle; light on 06.00 h) and standard environmental conditions (ambient temperature 20-22°C, humidity 55–60%) for at least 1 week before the commencement of experiments. Rat chow and tap water were available ad libitum. All surgery and experimental procedures were done during the light cycle and were approved by the Animal Ethics Committee of The Second University of Naples. **Animal care was in compliance with the IASP and European Community (E.C. L358/1 18/12/86) guidelines on the use and protection of animals in experimental research**. All efforts were made to reduce both animal numbers and suffering during the experiments.

### Neuropathic pain

The spared nerve injury model of neuropathic pain was induced according to the method used by Decosterd and Woolf [58]. Rats were anaesthetised with sodium pentobarbital (50 mg/kg, i.p.). The sciatic nerve was exposed at the level of its trifurcation into sural, tibial and common peroneal nerves. The sural and common peroneal nerves were ligated tightly then transected just distal to the ligation, leaving the tibial nerve intact. Sham rats were anaesthetised, the sciatic nerve was exposed at the same level, but not ligated. Thirteen days after surgery sham and SNI rats underwent the surgical preparation for intra-VL PAG microinjection (see below paragraph) and the day after (14th after SNI) tail flick tests coupled with single unit extracellular recording experiments were carried out. Sham and SNI animals were sacrificed for immunohistochemistry, RT-PCR and western blot analysis at 14th after SNI or sham surgery.

### Surgical preparation for intra-VL PAG microinjection

For in vivo studies (formalin test and single unit electrophysiological recordings associated to tail flick) rats were implanted with a guide cannula to perform direct intra-VL PAG administrations. Briefly rats were anaesthetised with pentobarbital (50 mg/kg, i.p.) and a 26-gauge, 12 mm-long stainless steel guide cannula was stereotaxically lowered until its tip was 1.5 mm above the VL PAG by applying coordinates from the atlas of Paxinos and Watson [59] (A: -7.8 mm and L: 0.5 mm from bregma, V: 4.3 mm below the dura) controlateral to peripheral formalin injection or the sciatic nerve injury. The cannula was anchored with dental cement to a stainless steel screw in the skull. We used a David Kopf stereotaxic apparatus (David Kopf Instruments, Tujunga, CA, USA) with the animal positioned on a homeothermic temperature control blanket (Harvard Apparatus Limited, Edenbridge, Kent).

Direct intra-VL PAG administration was conducted with a stainless steel cannula connected by a polyethylene tube to a SGE 1-μl syringe, inserted through the guide cannula and extended 1.5 mm beyond the tip of the guide cannula to reach the VL PAG (Figure 2A). A group of rats were implanted intentionally 1 mm outside the VL PAG serving as placement controls in order to verify the specificity of the effect of the microinjection site (Figure 2A). Volumes of 200 nl drug solution, or vehicle, were injected into the VL PAG over a period of 60 sec and the injection cannula gently removed 2 min later.

### In vivo experiment organization

In vivo experiments have been carried out 24 hours after the surgery for guide cannula implantation.

Formalin test has been carried out in awake rats while in vivo single unit electrophysiological recording associated to tail flick in sham and SNI rats has been performed on anaesthetized animals. Sham and SNI rats were undergone to cannula implantation at the 13th day after sham or SNI surgery in order to perform single unit electrophysiological recording associated to tail flick at the 14th day.

### Formalin test

Formalin-induced pain is a widely used test for persistent pain [60]. Fifty microliters of formalin (5%) or saline solution (0.9% NaCl) were injected subcutaneously into the dorsal surface of the hind paws of awake rats using a 30-gauge needle. Nociceptive responses were divided into two phases: an initial early short phase (0–7 min) caused by a primary afferent discharge produced by the stimulus, followed by a quiescent period and a second prolonged phase (15–60 min) of tonic pain (late phase). Each rat was placed in a plexiglass testing chamber to acclimatize for 30 min. A mirror was placed at a 45° angle under the cage to allow a full view of the hind paws. Rats which were undergone to formalin test received vehicle, 0.05% dimethyl sulfoxide, DMSO, in artificial cerebrospinal fluid (ACSF, composition in mM: KCl 2.5; NaCl 125; MgCl_2_ 1.18; CaCl_2_ 1.26) or MMPIP (5 μg) into the VL PAG 10 min before formalin. Immediately following formalin injection, rats were observed for 60 min by experimenter blind to the treatment. Paw lifting – the injected paw was lifted off the cage floor, paw licking – the injected paw was licked or bitten, were considered as pain behaviour. The time spent lifting or licking the injected paw was recorded every 5 min during the 60 min after the formalin injection. Data are expressed in min as mean ± SEM of the amount of time spent lifting and licking the injected hind paw.

### Single unit extracellular recordings associated to tail flick

Single unit extracellular recordings associated to tail flick have been carried out in the RVM while microinjecting MMPIP (5 μg/0.2 μl) or respective vehicle (0.05% DMSO in ACSF, 0.2 μl) into the VL PAG in sham and SNI rats. Indeed previous studies have shown the presence of output neurons from VL PAG projecting to RVM neurons [61,62]. 20–24 hrs after the guide cannula implantation, anaesthesia was induced with pentobarbital (50 mg/kg, i.p.) and maintained with a continuous infusion of propofol (5–10 mg/kg/h, i.v.). A thermal stimulus was elicited by a radiant heat source of a tail flick unit (Ugo Basile, Varese, Italy), focused on the rat tail approximately 3–5 cm from the tip. The intensity of the radiant heat source was adjusted to 50 mW (corresponding to 50 mJ per sec) at the beginning of each experiment in order to elicit a constant tail flick latency. Tail flicks were elicited every 5 min for at least 15 min prior to microinjecting the drug or its vehicle into the VL PAG. A glass-insulated tungsten filament electrode (3–5 MW) (FHC Frederick Haer & Co., ME, USA) was lowered into the RVM using the following stereotaxic coordinates: 2.8–3.3 mm caudal to lambda, 0.4–0.9 mm lateral and 8.9–10.7 mm depth from the surface of the brain [59] (Figure 2B). RVM noxious stimuli-responding neurons were identified by the characteristic OFF cell pause and ON cell burst of activity immediately prior to tail flick responses [47]. The recorded signals were amplified and displayed on both analogue and a digital storage oscilloscope to ensure that the unit under study was unambiguously discriminated throughout the experiment. Signals were sampled by a CED 1401 interface (Cambridge Electronic Design Ltd., UK) and analyzed by Spike2 window software (CED, version 4) to create peristimulus rate histograms on-line and to store and analyse digital records of single-unit activity off line. The configuration, shape, and height of the recorded action potentials were monitored and recorded continuously using Spike2 software for on-line and off-line analyses. Once an ON or OFF cell was identified from its background activity, we optimised spike size before all treatments. This study only included neurons whose spike configuration remained constant and could clearly be discriminated from the background activity throughout the entire experiment. By doing so, we were able to determine the activity of only one neuron. In each rat, the activity of only a single neuron was recorded before and after vehicle or drug administration. Ongoing and tail flick-related activity of the OFF cells was recorded before and after the VL PAG microinjection of vehicle or MMPIP in shams and SNI rats 14 days after the surgical procedure for neuropathic pain induction. For each OFF neuron the ongoing activity was obtained by averaging the firing rate (spikes/sec) for 50 sec before the tail flick trials (carried out every 5 min). The latency to the onset of the pause (time between the onset of heat application and the last action potential) and the duration of the tail flick-related pause (the time elapsing between the pause onset and the first action potential following tail flick) of the OFF cells were also quantified. Moreover, the peak height of the tail flick-related burst (spikes/sec) and the onset of the ON cell burst (the time elapsing between the onset of heat application and the increase in the frequency rate, which was at least twofold higher than its baseline) were quantified for the ON cells. At the end of the experiment, a volume of 200 nl of neutral red (0.1%) was injected into the VL PAG 30 min before killing the rats with a lethal dose of urethane. Rats were then perfused intracardially with 20 ml phosphate buffer solution (PBS) followed by 20 ml of 10% formalin solution in PBS. The brains were removed and immersed in a saturated formalin solution for 2 days. After fixation, the microinjection and recording sites were identified (Figure 2B). The injection sites were ascertained using two consecutive sections (40 μm), one stained with cresyl violet to identify the VL PAG, and the other unstained to determine dye spreading. The recording site was marked with a 20 μA DC current applied for 20 sec immediately prior to the end of the electrophysiological recordings. Only the data from microinjection and drug diffusion sites located within the VL PAG and those from the recording sites in RVM neurons were included in the results. Groups of 10–12 rats per each treatment have been used in order to have at least 5–6 recordings for ON and OFF cells with each animal being used for a single cell recording.

Rats received 14 days after surgery a single intra VL PAG microinjection of vehicle or MMPIP and were divided as follows:

1) Sham and SNI rats receiving an intra-VL PAG microinjection of vehicle,

2) Sham and SNI rats receiving intra-VL PAG microinjection of MMPIP.

3) SNI rats (n = 7) receiving microinjection of MMPIP intentionally 1 mm outside the VL PAG

### mRNA extraction and reverse transcriptase-PCR

The VL PAG was homogenized and total RNA extracted using an RNA Tri-Reagent (Molecular Research Center Inc.) according to the manufacturer’s protocol. The extracted RNA was subjected to DNase I treatment at 37°C for 30 min. The total RNA concentration was determined using a UV spectrophotometer. The mRNA levels of the gene for mGluR7 were measured by reverse transcriptase (RT)-PCR amplification. Sequences for rat mGluR7 mRNAs from GenBank (DNASTAR Inc.) were used to design primer pairs for RT-PCRs (OLIGO 4.05 software, National Biosciences Inc.). Each RT-PCR was repeated at least four times by an experimenter who was blind to the treatment. A semiquantitative analysis of mRNA levels was performed by the Gel Doc 2000 UV System (Bio-Rad). The measured mRNA levels were normalized with respect to ribosomial subunit 18S, chosen as housekeeping gene, and the gene expression values were expressed as mean of arbitrary units ± SEM.

### Western blot

The VL PAG was first minced into small pieces with a blender, and then suspended in lysis buffer (4% SDS, 20% glycerol, 10% 2-mercaptoethanol, 0.004% bromophenol blue, Tris–HCl, pH 6.8, containing 6 M urea, 50 μM Na3VO4, 50 μM PMSF, Sigma) for protein extraction. Total protein concentration was determined. Each sample was loaded, electrophoresed in an 8% polyacrylamide gel, and electroblotted onto a nitrocellulose membrane. To detect mGluR7, a rabbit polyclonal primary antibody (Santa Cruz Biotechnology, Inc) at 1:500 dilution was used. Following incubation, sections were washed and reincubated with secondary antibody solution (donkey anti-goat, IgG-HRP1:5000; Santa Cruz Biotechnology Inc). GE Healthcare enhanced chemiluminescence (ECL) substrate (Pierce) was used. Protein levels were normalized with respect to the signal obtained with β-tubulin monoclonal antibodies (Santa Cruz Biotechnology; 1:1000 dilution) chosen as housekeeping protein, and the protein expression values were expressed as mean of arbitrary units ± SEM.

### Immunohistochemistry

Sham and SNI rats were anaesthetized with pentobarbital (50 mg/kg, i.p.) and transcardially perfused with saline solution followed by 4% paraformaldehyde in 0.1 M phosphate buffer. The brain was removed, post fixed for 3 hours in the perfusion fixative, cryoprotected for 72 hours in 10, 20 and 30% sucrose in 0.1 M phosphate buffer and frozen in O.C.T. embedding compound. Transverse sections (15 μm) were cut using a cryostat and those containing the whole PAG were thaw-mounted onto glass slides. Sections were subsequently incubated for 1 day at room temperature in a humid chamber with the respective polyclonal antibodies (all diluted in specific block solution). All sections were processed for goat anti-vesicular glutamate transporter-1 (VGluT1) (1: 100, Santa Cruz, USA), goat anti-vesicular GABA transporter (VGAT) (1:100, SySy, Germany), rabbit-anti mGlu7 receptor (1:100, Santa Cruz, USA) and anti-synaptophysin (rabbit polyclonal SySy, Germany). **F**ollowing incubation, sections were washed and incubated for 3 hours with secondary antibody solution (donkey anti-goat, or donkey anti-rabbit IgG-conjugated Alexa FluorTM 488 and 568; 1:1000; Molecular Probes, USA). Slides were washed, cover-slipped with Vectashield mounting medium (Vector Laboratories, USA) and visualized under a Leica fluorescence microscope. Negative control by using secondary antibodies alone did not reveal any positive staining. Toth and Mezey [63] procedure has been used for the double labelling.

### Drugs

6-(4-methoxyphenyl)-5-methyl-3-pyridin-4-ylisoxazolo[4,5-c]pyridin-4(5H)-one (MMPIP) was purchased from Tocris Bioscience (Bristol, UK) and was dissolved in 0.05% dimethyl sulfoxide (DMSO) in ACSF (vehicle) on the day of the experiment. The dose of MMPIP has been chosen according to in vivo studies using intra-cerebral microinjections which have shown to antagonize AMN082 effects [64,65].

### Data analysis and statistics

All data are given as means ± SEM. For behavioural and electrophysiology experiments mixed design two-way ANOVA has been used to analyze statistical differences between the different groups of rats. Paired t test has been used to compare post-injection versus pre-injection values. Two-tailed unpaired Student’s t-tests was used for biomolecular analysis and protein quantification. P values < 0.05 were considered statistically significant.

## Competing interests

The authors declare that they have no competing interests.

## Authors’ contributions

EP has organized, conceptualized and written the manuscript. IM and MS have helped in analyzing the data. LL and MEG have performed the experiments of immunohistochemistry. SB have performed in vivo experiments. SB has also performed the statistical analysis. GB and FR have performed the biomolecular and western blot experiments. VdN and SM have contributed to draft of the paper. All authors have read and approved the final manuscript.
